# Ribosome Incorporation Transdifferentiates Chick Primary Cells and Induces Their Proliferation by Secreting Growth Factors

**DOI:** 10.3390/jdb13020019

**Published:** 2025-06-01

**Authors:** Shota Inoue, Arif Istiaq, Anamika Datta, Mengxue Lu, Shintaro Nakayama, Kousei Takashi, Nobushige Nakajo, Shigehiko Tamura, Ikko Kawashima, Kunimasa Ohta

**Affiliations:** 1 Department of Stem Cell Biology, Graduate School of Systems Life Sciences, Kyushu University, 744 Motooka, Nishi-Ku, Fukuoka 819-0395, Japan; inoue.shota.090@s.kyushu-u.ac.jp (S.I.); istiaqarif@gmail.com (A.I.); anudhaka1419@gmail.com (A.D.); nkym.str.shin-naka@hotmail.com (S.N.);; 2 IntegriCulture, 8-1 Kawada-Cho, Shinjuku-ku, Tokyo 162-0054, Japan; lumengxue.11@gmail.com (M.L.); ikko@integriculture.com (I.K.); 3 Department of Biology, Faculty of Science, Kyushu University, 744 Motooka, Nishi-Ku, Fukuoka 819-0395, Japan; nakajo.nobushige.580@m.kyushu-u.ac.jp; 4 Department of Molecular Cell Biology, Faculty of Arts and Science, Kyushu University, 744 Motooka, Nishi-Ku, Fukuoka 819-0395, Japan; stamura@artsci.kyushu-u.ac.jp; 5 Department of Stem Cell Biology, Faculty of Arts and Science, Kyushu University, 744 Motooka, Nishi-Ku, Fukuoka 819-0395, Japan

**Keywords:** chick primary cells, ribosomes, cell proliferation, cultured meat

## Abstract

Previously, we reported that mammalian cells, specifically human dermal fibroblasts (HDFs), could be transdifferentiated by lactic acid bacteria (LAB). Later, we observed that HDFs incorporated LAB-derived ribosomes, forming the ribosome-induced cell clusters (RICs) and transdifferentiating into cells derived from all three germ layers. Based on this insight, we hypothesized that incorporating ribosomes into non-mammalian cells could reveal the universality of this mechanism and open the door to commercial applications. Our current study demonstrates that ribosome incorporation can transdifferentiate chick primary muscle-derived cells (CMCs) into adipocytes, osteoblasts, and chondrocytes. Furthermore, the culture medium supernatant from ribosome-incorporated CMCs was found to significantly enhance CMC’s proliferation. RNA-seq analysis revealed that RICs-CMC exhibit increased expression of genes related to multi-lineage cell growth. In addition, we developed a novel technological shift in meat production—the “CulNet System”—which replicates organ interactions within mechanical systems for cell-cultured meat production. While significant efforts are still required to implement this technology in a cost-effective manner, we believe that combining the “CulNet System” with ribosome-incorporated multipotent cells that have prolonged culture capability could substantially improve the scalability and cost-effectiveness of cultured chicken meat production. This report highlights a promising approach for cell-culture-based meat production, offering a sustainable alternative to traditional methods.

## 1. Introduction

The consumption of livestock meat is a prominent feature of human diets worldwide. However, its consumption is associated with numerous global challenges, including pollution, resource depletion, and adverse environmental impacts [1]. For instance, methane gas released from cattle burping significantly contributes to these environmental burdens [2]. Methane has a greenhouse effect 23 times greater than that of carbon dioxide [3].

Globally, methane emissions from cattle and other livestock are estimated to be the equivalent amount of approximately 3 billion tons of carbon dioxide annually, accounting for about 5% of total global greenhouse gas emissions [4]. Furthermore, inefficient animal husbandry, combined with rapid economic development and population growth in developing countries, has led to the widespread depletion of essential resources such as water, feed, and grazing land. Specifically, producing 1 kg of beef is estimated to require 24 kg of feed and tens of thousands of liters of water [5]. To address these challenges, various biotechnology companies are actively researching and developing cell-cultured and plant-based meats as sustainable and environmentally friendly protein sources.

Previously, we reported that incorporating lactic acid bacteria (LAB) into human dermal fibroblasts (HDFs) induced the formation of multipotent cell clusters capable of differentiating into cells derived from all three germ layers [6,7]. We subsequently identified ribosomes within LAB as key factors facilitating the acquisition of multipotency through transdifferentiation, and similar findings were observed in HDFs and mouse embryonic fibroblasts (MEFs) [8,9,10]. Additionally, we demonstrated that ribosomes exhibit transdifferentiating effects on adult mouse skin fibroblasts, human cancer cell lines, and glioblastoma stem-like cells [11,12,13,14,15]. However, the universality of this phenomenon in primary cells from other species has yet to be elucidated. To explore this, we investigated whether chick primary muscle-derived cells (CMCs) could acquire multipotency and differentiate into other cell types upon incorporation of ribosomes.

The production of cell-cultured meat is being pursued as a potential solution to environmental and resource-related challenges [16]. However, the high production costs, compared to livestock meat, have hindered its widespread adoption. These elevated costs are primarily attributed to the reliance on multiple growth factors in the culture medium [17]. Moreover, the field of cultured meat remains in its early stages, and technologies for large-scale culturing of cells, particularly those with muscle-like tissue organization, are still under development. Despite these challenges, progress continues to be made annually, with the goal of optimizing the cost and productivity of cell-cultured meat [18,19]. To this end, the bio-venture company IntegriCulture has developed a promising approach to address some of these challenges. The company’s CulNet System simulates the interactions between organs within an organism [20]. In this system, various cell types are cultured in individual containers, and the supernatant is collected and circulated, similar to the function of blood, to supply nutrients to the target cells. This approach eliminates the need for costly growth factors and has led to significant cost reductions.

Nevertheless, further reduction in production costs remains a critical challenge for the practical implementation of cultured meat. In fact, various studies have been conducted aimed at replacing conventional growth factors and improving the efficiency of cell culture. For example, recombinant porcine FGF1 purified from *Escherichia coli* (*E. coli*) has been used as a substitute for traditional growth factors, and research into serum-free media that do not rely on animal-derived growth factors has also been carried out [21,22]. Additionally, conditioned medium derived from the rat liver epithelial cell line RL34 has been reported to promote the proliferation of other cell types [23,24]. Building upon these prior studies, we hypothesized that the application of ribosome-incorporation technology to induce the formation of multipotent cell clusters could enhance production efficiency within the CulNet System. Specifically, we speculated that ribosome-incorporated cells might acquire stem cell-like characteristics and secrete growth factors and other beneficial substances extracellularly, thereby contributing to increased production efficiency and reduced costs in the CulNet System. To evaluate this hypothesis, we investigated whether ribosome-incorporated cells secrete growth factors into the culture supernatant. Cells were cultured using the collected supernatant, and their proliferation rates were evaluated. As a result, cell proliferation was significantly enhanced when cultured with supernatant derived from ribosome-incorporated cells. These findings suggest that combining ribosome-incorporated cell technology with the CulNet System could improve production efficiency and reduce costs in cultured meat production.

## 2. Materials and Methods

### 2.1. Isolation of Chick Muscle-Derived Cells

To isolate chick muscle-derived cells, fertilized chicken eggs obtained from Yamagishi (Wakayama, Japan) were incubated at 37 °C and 60% humidity for 12 days in an incubator (Showa Furanki, Saitama, Japan). The eggs were then cracked open, and chicken thigh muscle tissues were collected in Dulbecco’s Modified Eagle Medium (DMEM) containing 1% Penicillin-Streptomycin-Amphotericin B (PSA) solution. The tissues were mechanically dissociated using a 10 mL syringe plunger and filtered through a 100 μm cell strainer to isolate the cells, using DMEM with 1% PSA. The resulting cell suspension was washed with DMEM containing 1% PSA by centrifugation at 300× *g* for 5 min. After removing the supernatant, the cell pellet was resuspended in DMEM with 10% Fetal Bovine Serum (FBS) and 1% PSA, and plated in 10 cm culture dishes. All procedures were performed under sterile conditions on a clean bench to prevent contamination.

### 2.2. Isolation and Purification of Ribosomes

Tetra-(His)6-tagged ribosomes were isolated from *E. coli* JE28, a strain kindly provided by the Department of Cell and Molecular Biology, Uppsala University, Sweden. Ribosome isolation and purification from *E. coli* JE28 were performed as previously described [8,25]. Briefly, 1% of pre-cultured exponential-phase bacteria were inoculated into fresh LB medium and incubated until cells reached the exponential phase (OD_600_ 0.6–0.8). The culture was chilled immediately and collected by centrifugation at 8000 revolutions per minute (RPM) for 5 min at 4 °C. The pellet was resuspended in 1× TMAK buffer (10× buffer: 200 mM Tris-HCl, pH 7.8; 100 mM MgCl_2_; 300 mM NH_4_Cl; 1.5 M KCl) with 100 mM PMSF, then disrupted by sonication. Cell debris was removed by centrifugation at 10,000 RPM for 10 min at 4 °C. The supernatant was collected and incubated overnight at 4 °C with cOmplete His-tag purification beads (Roche, Basel, Switzerland). Ribosomes were purified using a cOmplete His-tag purification column (Roche, Basel, Switzerland). Final purification was carried out using Amicon Ultra 100-kDa filters (Merck, Darmstadt, Germany). Protein concentrations were determined using a protein assay kit (Bio-Rad, Hercules, CA, USA) and adjusted to the desired concentration with 1× (Phosphate-Buffered Saline) PBS.

### 2.3. Ribosome-Induced Cell Clusters (RICs) Formation of CMCs

Chick muscle-derived cells were cultured until they reached 90% confluency, then trypsinized (Sigma-Aldrich, St. Louis, MO, USA). Trypsinized cells were resuspended in 500 μL ESGRO (Sigma-Aldrich), and 40 μg of purified ribosomes were added to the cell suspension, adjusting the concentration to 1 × 10^5^ cells per well in a 4-well dish. A control plate was prepared in parallel without ribosomes. The detailed strategy for optimizing ribosome quantity in relation to cell number has been previously discussed [11]. The following day, an additional 500 μL of ESGRO was added to each well, and half of the medium was replaced every 2–3 days. These ribosomes induced cell clusters of chicken muscle-derived cells were termed as RICs-CMC in the following sections.

### 2.4. Immunocytochemistry

Chick muscle-derived cells and ribosomes were co-cultured on poly-L-lysine (Sigma-Aldrich)-coated glass slides for 2 days. For immunocytochemistry, cells were fixed with 4% paraformaldehyde (PFA) for 15 min at room temperature (RT), washed three times for 10 min with 1× PBS containing 0.1% Triton X-100 (Sigma-Aldrich), and blocked with 5% skim milk in 1× PBS containing 0.1% Triton X-100 for 1 h at RT. Cells were incubated overnight at 4 °C with a primary antibody (Mouse anti-His tag, 1:200, ab18184, Abcam, Cambridge, UK). After washing with 1× PBS containing 0.3% Triton X-100, cells were incubated with a secondary antibody (Goat anti-Mouse IgG2a, 1:800, 1080-02, SouthernBiotech, Birmingham, AL, USA) for 2 h at RT in the dark (Supplementary Table S1). After washing, cells were stained with Hoechst (1:500, H3570, Invitrogen, Waltham, MA, USA) for 15 min at RT in the dark. The slides were mounted and imaged using a TCS SP8 STED microscope (Leica, Wetzlar, Germany).

### 2.5. Alkaline Phosphatase Staining

RICs cultured for 1 week were assessed for alkaline phosphatase (AP) activity using the Purple-Color AP Staining Kit (System Biosciences, Palo Alto, CA, USA) following the manufacturer’s instructions. Briefly, RICs were fixed with the provided fixation solution for 10 min at RT, washed twice with 1× PBS, and incubated with AP staining solution for 30 min at RT in the dark. After incubation, clusters were washed twice with 1× PBS, and images were captured using a Leica microscope (DM IL LED Fluorescence Inverted Laboratory Microscope).

### 2.6. Transdifferentiation of RICs-CMC

For transdifferentiation assays, 10 to 100 RICs-CMC were collected 11 days after ribosome incorporation and transferred to the appropriate differentiation induction medium. Adipocyte differentiation was induced using Adipocyte Differentiation Basal Medium StemPro^®^ (Gibco, Carlsbad, CA, USA), osteoblast differentiation using Osteoblast Differentiation Basal Medium StemPro^®^ (Gibco), and chondrocyte differentiation using Chondrocyte Differentiation Basal Medium StemPro^®^ (Gibco). Cells were cultured in the differentiation medium for 14 days (adipocytes) or 21 days (osteoblasts and chondrocytes). Differentiated cells were stained with Oil Red O (Sigma-Aldrich), Alizarin Red S (Sigma-Aldrich), or Alcian Blue (Nacalai Tesque, Kyoto, Japan), respectively. The stained images were analyzed by ImageJ (version 1.53).

### 2.7. Proliferation Assay

Chick muscle-derived cells were cultured to 90% confluency, trypsinized, and resuspended in 500 μL DMEM containing 40 μg of purified ribosomes. A control plate was prepared without ribosomes. After 24 h, the medium was removed, and cells were washed three times with 1× PBS. Fresh medium (1 mL) was added and cultured for 1 week without changing the medium. After 1 week, the culture supernatant was collected, and fresh trypsinized chick muscle-derived cells were suspended in 1 mL of the collected supernatant, adjusting to 1 × 10^5^ cells per well in a 4-well dish. Half of the medium was replaced every 3 days. Cell counts were performed using a hemocytometer on days 1, 3, and 5.

### 2.8. RNA-Sequencing (RNA-Seq)

Control chick muscle-derived cells and ribosome-incorporated chick muscle-derived cells (RICs-CMC) were prepared for RNA-seq according to the sample preparation guidelines from Active Motif. Briefly, control chick muscle-derived cells (1.0 × 10^6^ cells/well) and RICs-CMC (1.0 × 10^6^ cells/well, 130 µg ribosomes) were cultured in ESGRO medium for 14 days. Replicates were prepared to reach a total of 2.0 × 10^6^ cells per condition. Control cells were gently scraped from the culture surface, while RICs-CMC were collected in their floating state. Both samples were centrifuged at 800× *g* for 5 min at 4 °C to remove residual culture medium. The cell pellets were then resuspended in chilled PBS and spun down again under the same conditions. The resulting pellets were flash-frozen in liquid nitrogen and stored at −80 °C until RNA isolation.

Next, RNA libraries were prepared from the purified RNA using the TruSeq Stranded mRNA Sample Preparation kit (Illumina). RNA-sequencing (150 nt paired end reads) was performed utilizing a NovaSeq 6000 (Illumina), following the manufacturer’s guidelines. FastQ reads generated from the samples were processed using the fastp tool (version 0.23.4) to trim low-quality regions (with a Phred quality score cutoff of Q = 30) and remove adapter sequences [26]. The trimmed paired-end reads were aligned to the GRCg7b reference genome (bGalGal1.mat.broiler.GRCg7b, GCA_016699485.1) using the HISAT2 program (version 2.2.1) [27]. Gene-level read counts were extracted using featureCounts (version 2.0.1) with the corresponding GTF file (Ensembl release-111) from the Ensembl genome browser [28]. The read counts were then normalized by DEseq2 (version 1.40.2) and Transcripts Per Million (TPM) [29,30].

### 2.9. Statistical Analysis

Statistical analysis was performed using R (version 4.3.1). Data were analyzed using ANOVA followed by the Tukey–Kramer test for multiple comparisons. Statistical significance was determined as follows: *p* < 0.05 was considered statistically significant.

## 3. Results

### 3.1. Incorporation of Ribosomes into Chick Muscle-Derived Cells Forms Cell Clusters

**Figure 1:**
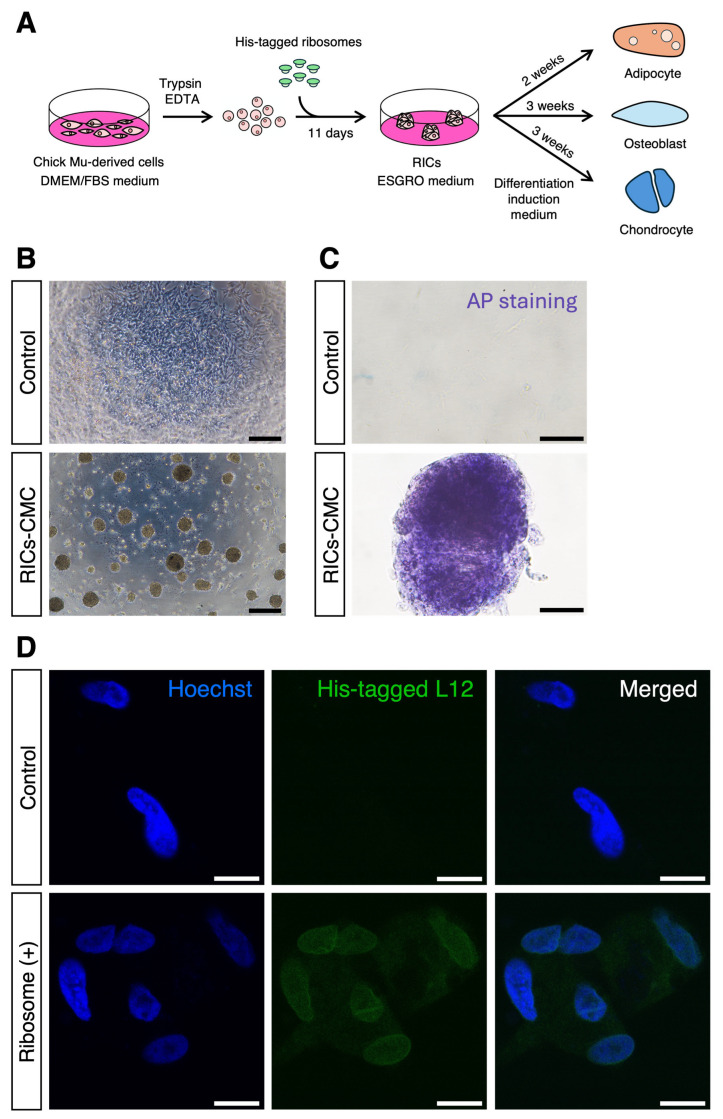
Ribosome induces cell cluster formation in CMCs. (**A**) Illustration of the ribosome incorporation method. (**B**) Cluster formation of CMCs incorporating ribosomes; 40 µg of ribosomes were added to 1.0 × 10^5^ cells per well in a 4-well dish. Cluster formation was recorded 1 day after incorporation. Scale bars: 300 µm. (**C**) AP staining of RICs-CMC; staining was performed 6 days after incorporation. Scale bars: 50 µm. (**D**) Localization of exogenous ribosomes in RICs-CMC. Immunocytochemistry was performed using an anti-6× His antibody, which recognizes the His-tagged L-12 ribosomal protein to confirm ribosome incorporation into RICs-CMC on day 2 after incorporation. Scale bars: 10 µm.

Previously, we reported that the incorporation of ribosomes into HDFs and MEFs resulted in the formation of cell clusters [8,9,10]. However, this phenomenon has not yet been validated in non-mammalian cells. In this study, we used avian chick primary muscle-derived cells to investigate the effects of ribosome incorporation. CMCs were cultured, treated with trypsin, mixed with 40 μg of purified ribosomes, and co-cultured in ESGRO medium (Figure 1A). This treatment resulted in the formation of ribosome-induced cell clusters (RICs-CMC) (Figure 1B).

To assess the potential multipotency of these clusters, we performed AP staining on the RICs-CMC, which resulted in purple staining, suggesting that the clusters possess characteristics indicative of multipotent cells (Figure 1C). Additionally, immunocytochemistry was performed to confirm the intracellular localization of the incorporated ribosomes. Ribosomes were tagged with a His-tag on the L12 protein, and the presence of the His-tag was detected using an anti-His antibody. Interestingly, the His-tag signal was observed not only in the cytoplasm but also in the nucleus, which aligns with previous findings (Figure 1D) [8,11,13].

### 3.2. RICs-CMC into Mesodermal Lineage Cells In Vitro

**Figure 2:**
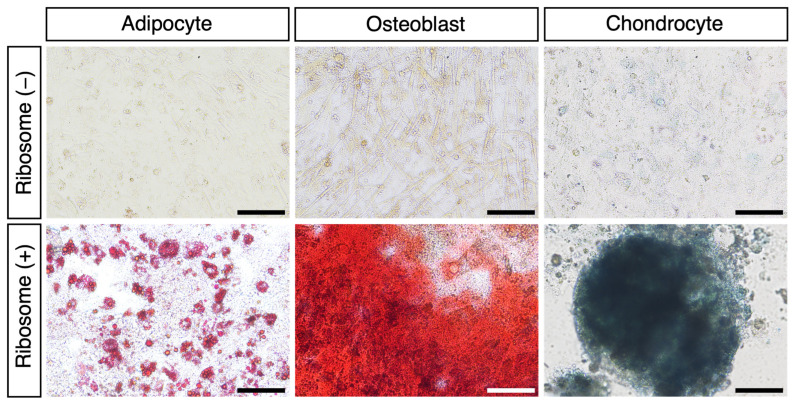
Ribosome-incorporated CMCs differentiate into adipocytes, osteoblasts, and chondrocytes. For each condition, 10–100 clusters were transferred to a specific differentiation induction medium (per well of a 24-well dish) and cultured for 14 days (adipocytes) or 21 days (osteoblasts and chondrocytes). Differentiated adipocytes, osteoblasts, and chondrocytes were stained with Oil Red O, Alizarin Red S, or Alcian Blue, respectively. Optical microscope images are shown. Scale bars: 100 µm.

To investigate the differentiation potential of RICs-CMC, we cultured the cell clusters in specific differentiation induction media. After 11 days of culturing, the RICs-CMC were transferred to differentiation media to induce adipocyte differentiation (14 days), osteoblast differentiation (21 days), and chondrocyte differentiation (21 days). The differentiated cells were stained using appropriate staining reagents, and the results confirmed successful differentiation into adipocytes, osteoblasts, and chondrocytes, respectively (Figure 2 and Supplementary Table S2: Adipogenesis; 17 ± 1.7%, Osteogenesis; 81 ± 4.9%). These results indicate that ribosome-incorporated CMCs are capable of differentiating into various mesodermal lineage cells in vitro.

### 3.3. Cell Culture Supernatant from Ribosome-Incorporated CMCs Enhances Cell Proliferation

**Figure 3:**
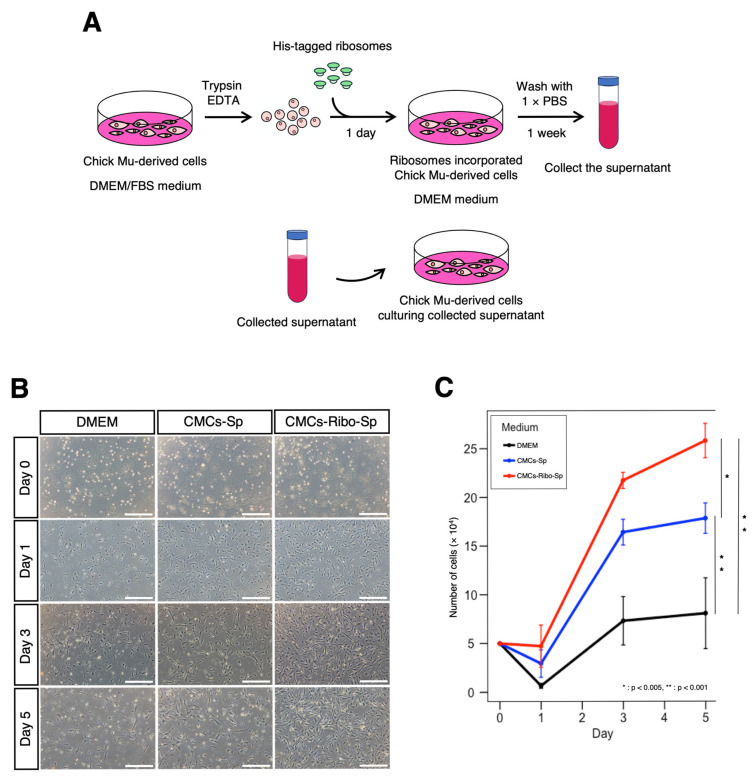
Ribosome-incorporated CMC culture supernatants enhance cell proliferation. (**A**) Illustration of the ribosome incorporation method. (**B**) CMC proliferation progress; CMCs were seeded at 5.0 × 10^4^ cells per well in a 24-well dish and cultured in the following media: DMEM (serum-free), CMCs-Sp (culture supernatant of CMCs), and CMCs-Ribo-Sp (culture supernatant of CMCs incorporated ribosomes). Optical microscope images are shown. Scale bars: 500 µm. (**C**) Cell growth was monitored daily until day 5, with cell counts plotted over time. Cells were counted using a hemocyter. Tukey’s multiple comparison test was used to compare cell counts (*n* = 4, *: *p* < 0.005, **: *p* < 0.001).

We hypothesized that ribosome-incorporated CMCs might exhibit stem cell-like characteristics, such as the secretion of growth factors into the extracellular environment. To test this hypothesis, we collected the culture supernatants from CMCs following ribosome incorporation and evaluated their effects on CMC proliferation (Figure 3A).

When cultured in serum-free DMEM, CMCs showed poor adhesion and minimal proliferation by Day 1, with little to no increase in cell number between Day 3 and Day 5. In contrast, CMCs cultured in supernatants from ribosome-free CMCs (CMCs-Sp) exhibited better adhesion and a higher proliferation rate than those cultured in DMEM. Notably, CMCs cultured in supernatants from ribosome-incorporated CMCs (CMCs-Ribo-Sp) showed similar adhesion to CMCs-Sp but exhibited a significantly higher proliferation rate thereafter. By Day 5, cell proliferation in the CMCs-Ribo-Sp group was markedly enhanced compared to the other conditions (Figure 3B,C and Supplementary Table S4). These findings suggest that CMCs that incorporate ribosomes secrete factors that promote cell growth.

### 3.4. Ribosome-Incorporated CMCs Express Several Cell Growth Factors

**Figure 4:**
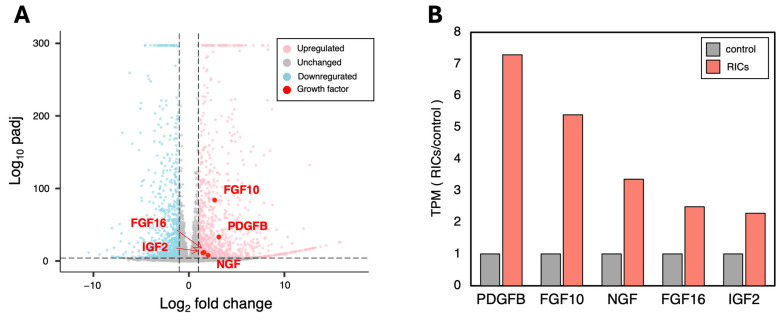
RNA-seq analysis reveals increased expression of various cell growth-related factors in RICs-CMC. (**A**) Differentially expressed genes (DEGs) analysis was performed. Five growth factor-related genes showed a particularly high increase in expression (Log2 fold change > 1, Log10 padj > 5). (**B**) Changes in expression levels of the top 5 genes, which exhibited the most significant fluctuations in expression. TPM-corrected values are shown (*n* = 2).

To investigate the specific growth factors released by CMCs incorporating ribosomes, we performed RNA-seq analysis. Differentially expressed gene (DEG) analysis revealed dynamic fluctuations in gene expression. Among the genes with significantly increased expression, we identified the top five growth factors involved in cell proliferation: PDGFB, FGF10, NGF, FGF16, and IGF (Figure 4A).

Further analysis of the TPM-corrected expression levels revealed a 2- to 7-fold increase in the expression of each of these genes in the ribosome-incorporated CMCs compared to control CMCs (Figure 4B). These results suggest that ribosome-incorporated CMCs release a variety of cell growth factors that may contribute to the observed enhanced cell proliferation.

## 4. Discussion

In this study, we demonstrated that the incorporation of *E.coli*-derived ribosomes into chicken muscle-derived cells (CMCs) resulted in the formation of multipotent-like cell clusters (RICs-CMC), which were capable of differentiating into mesodermal lineage cells, including adipocytes, osteoblasts, and chondrocytes. Furthermore, it was revealed that the conditioned medium derived from ribosome-incorporated cells significantly enhanced the proliferation of CMCs. RNA-seq analysis indicated increased expression of cell proliferation factors such as PDGFB, FGF10, NGF, FGF16, and IGF, suggesting that ribosome incorporation promotes the secretion of extracellular factors that stimulate cell proliferation. These findings suggest that ribosome-mediated transdifferentiation is not limited to mammalian cells but can be extended to a broader range of species, highlighting its potential applications in the cultured meat production industry.

Previously, we reported that ribosome incorporation induced multipotency in mammalian cells, including HDFs and MEFs [8,9,10]. However, the universality of this phenomenon in non-mammalian cells remained unclear. In this study, we sought to verify the universality of ribosome-mediated transdifferentiation using avian cells, specifically CMCs. We confirmed that ribosome incorporation into CMCs resulted in the formation of stem cell-like clusters. Additionally, we demonstrated that RICs-CMC could differentiate into mesodermal lineage cells, including adipocytes, osteoblasts, and chondrocytes, as assessed through Oil Red O staining, Alizarin Red S staining, Alcian Blue staining, and image analysis. Furthermore, quantitative analyses using RT-PCR and RT-qPCR were conducted to detect lineage-specific marker genes (adipocytes: PPARG, LPL; osteoblasts: RUNX2, COL1A2, SPP1; chondrocytes: COL2A1, COL10A1, Supplementary Table S1) [31,32,33]. Despite using multiple primer sets for each target gene, no lineage-specific marker genes were detected. The CulNet System is designed to provide a self-sustaining cell culture environment accessible to farmers [34]. However, the chicken strains raised on farms may differ from the White Leghorn strains commonly used in basic research, possibly leading to genomic differences and the inability to detect marker genes. Nevertheless, our results demonstrate that this phenomenon can be extended to avian cells, representing an important step toward broadening the applicability of ribosome-mediated transdifferentiation.

RNA-seq analysis revealed dynamic changes in gene expression triggered by ribosome incorporation. Notably, the expression of genes associated with cell growth factors, such as PDGFB, FGF10, and IGF, was significantly upregulated. For example, FGF10 is a critical growth factor that plays an essential role in limb formation during chick embryogenesis [35]. Furthermore, additional factors are believed to contribute to the regulation of cell proliferation. The upregulation of these genes suggests that ribosome incorporation activates pathways that enhance cell proliferation. Furthermore, immunocytochemical analysis showed that the ribosomal protein L12 localized not only to the cytoplasm but also to the nucleus, hinting toward a potential role in regulating gene expression to enhance the secretion of cell growth factors. Further investigations using proteomics and metabolomics are necessary to gain deeper insights into the intracellular and extracellular changes induced by ribosome incorporation [36,37].

The findings of this study have significant implications for the cultured meat industry. One of the major challenges in cultured meat production is the high cost associated with growth factors and culture media [17]. As demonstrated in this study, CMCs that incorporated ribosomes secrete growth factors that promote cell proliferation, offering a novel strategy to address this challenge. Moreover, integrating the ribosomes into the CulNet System could strengthen the self-sustaining culture environment by enabling cells to produce the growth factors necessary for their proliferation. This integration is expected to improve production efficiency and reduce the overall cost of cultured meat production.

However, several challenges remain to be addressed. First, it is necessary to determine whether ribosome incorporation induces similar transdifferentiation effects in other non-mammalian species and cell types. Additionally, the long-term stability of ribosome-incorporated cells and the functionality of the secreted growth factors under practical conditions require thorough evaluation. Furthermore, safety assessments and regulatory compliance of this technology in food production are essential for its commercial application.

We clarified that by introducing ribosomes into the cells, we can promote the secretion of growth factors. However, these results were obtained using culture dishes, and in practice, there are limitations in scaling up and improving the quality for the industrial production of cultured meat. In the future, applying ribosomes to the CulNet System will be necessary to investigate the conditions for producing high-quality cultured meat on a large scale. Elucidating the molecular mechanisms underlying ribosome-mediated transdifferentiation is crucial for optimizing this technology and expanding its range of applications.

## 5. Conclusions

In this study, we demonstrated that incorporating ribosomes into CMCs induces the formation of multipotent-like cell clusters (RICs-CMC). These RICs-CMC were shown to differentiate into mesodermal lineage cells, including adipocytes, osteoblasts, and chondrocytes. Furthermore, the conditioned medium derived from ribosome-incorporated cells significantly promoted the proliferation of CMCs. RNA-seq analysis revealed the upregulation of genes encoding growth factors associated with cell proliferation, such as PDGFB and FGF10.

These findings suggest that ribosome-incorporation technology is not limited to mammalian cells but is also applicable to avian cells, where it facilitates the induction of multipotency and the secretion of growth-promoting factors. Importantly, the use of supernatant derived from ribosome-incorporated cells highlights the potential to construct a self-sustaining, cost-effective cell culture environment within the CulNet System.

Taken together, the integration of ribosome-incorporated cell technology with the CulNet System represents a promising approach to enhance production efficiency and reduce costs in cultured meat production. This strategy could contribute to the future development of sustainable protein production technologies.

## Data Availability

The data are available upon request to the corresponding author.

## Supplementary Materials

The following supporting information can be downloaded at: https://www.mdpi.com/article/10.3390/jdb13020019/s1, Supplementary Table S1: Antibodies used in Immunocytochemistry.; Supplementary Table S2: Statistical Analysis of Staining for differentiated Adipocyte and Osteoblast.; Supplementary Table S3: Counted Cell Number of Proliferation assay.; Supplementary Table S4: RT-PCR and RT-qPCR Primers.; Supplementary Table S5: RNA-sequencing analysis by DEseq2 tool.; Supplementary Table S6: RNA- Sequencing-Significant DEG analysis by TPM.; Supplementary Table S7: RNA- Sequencing-Significant DEG analysis by TPM for growth factor.
