# Supplementary material for: Ribosome Incorporation Transdifferentiates Chick Primary Cells and Induces Their Proliferation by Secreting Growth Factors
Source: J Dev Biol. 2025 Jun 1;13(2):19. doi: 10.3390/jdb13020019 (PMC12193757; doi:10.3390/jdb13020019)
Supplement: Supplementary file 1 [file jdb-13-00019-s001.zip › Table S1. Antibodies used in Immunocytochemistry.pdf]

**Supplementary Table S1: Antibodies used in Immunocytochemistry**

| Primary Antibody | Host | Dilution | Company |
|---|---|---|---|
| Anti-6X His tag® | Mouse | 1:200 | Abcam |

| Secondary Antibody | Host | Dilution | Company |
|---|---|---|---|
| Goat Anti-Mouse IgG2a, Human ads-FITC | - | 1:800 | SouthernBiotech |
