# Supplementary material for: Ribosome Incorporation Transdifferentiates Chick Primary Cells and Induces Their Proliferation by Secreting Growth Factors
Source: J Dev Biol. 2025 Jun 1;13(2):19. doi: 10.3390/jdb13020019 (PMC12193757; doi:10.3390/jdb13020019)
Supplement: Supplementary file 1 [file jdb-13-00019-s001.zip › Table S4. RT-PCR & RT-qPCR Primers.pdf]

**Supplementary Table S4: RT-PCR & RT-qPCR Primers**

| Gene | Forward | Reverse |
|---|---|---|
| GAPDH | GGTGGTGCTAAGCGTGTTA | CCCTCCACAATGCCAA |
| PPARG | CGAATGCCACAAGCGGAGAAGG | CTTGGCTTTGGTCAGCGGGAA |
| LPL | AGTGAAGTCAGGCGAAAC | ACAAGGCACCACGATT |
| RUNX2 | TCTCTGAACTCTGCACCAAGTC | GCTCGGAAGCACCTGAGAGG |
| COL1A2 | AAGGATGGTCGCAATG | GGTGGCTAAGTCTGAGGT |
| SPP1 | CAGAACAGCCGGACTTTC | CTTGCTCGCCTTCACCAC |
| COL2A1 | GGACCAGCAAGACGAAAGAC | ATATCCACGCCAAACTCCTG |
| COL10A1 | CCACCTGGATTCTCCACTGT | TGCCAACTTCTCCAGGTTCT |
